# Genetic variants of IFIH1 and DHX58 affect the chronicity of hepatitis C in the Chinese Han population

**DOI:** 10.7717/peerj.14740

**Published:** 2023-01-30

**Authors:** Peng Huang, Jing-Jing Wu, Jin-Wei Zhang, Yu-Qing Hou, Ping Zhu, Rong Yin, Rong-Bin Yu, Yun Zhang, Ming Yue, Wei Hou

**Affiliations:** 1 Department of Epidemiology, Center for Global Health, School of Public Health, Nanjing Medical University, Nanjing, China; 2 The State Key Laboratory of Virology, School of Basic Medical Sciences, Wuhan University, Wuhan, China; 3 The Department of Environmental Health, Yangzhou Center for Disease Control and Prevention, Yang-zhou, China; 4 Department of Anesthesiology, Nanjing Drum Tower Hospital, Nanjing, China; 5 Department of Medical Affairs, Jiangsu Provincial People’s Hospital, Nanjing, China; 6 Department of Thoracic Surgery, Jiangsu Key Laboratory of Molecular and Translational Cancer Research, Jiangsu Cancer Hospital & Nanjing Medical University Affiliated Cancer Hospital & Jiangsu Institute of Cancer Research, Nanjing, China; 7 Department of Infectious Diseases, The First Affiliated Hospital of Nanjing Medical University, Nanjing, China

**Keywords:** IFIH1, DHX58, Hepatitis C, Polymorphism, Chronicity

## Abstract

Hepatitis C remains a major public health problem in the world. The host immune system plays a key role in viral clearance. This study aimed to investigate the connection between retinoic acid-inducible gene I-like (RIG-I-like) receptor gene polymorphism and hepatitis C chronicity in the Chinese Han population. The current study genotyped three SNPs (IFIH1 rs10930046 and DHX58 rs2074158, rs2074160) to assess their association with the chronicity of hepatitis C virus (HCV) infection among 1,590 participants (590 spontaneous HCV clearance cases and 1,000 persistent infection patients). Our research shows that DHX58 rs2074158-G allele (dominant model: adjusted OR = 1.53, 95% CI [1.20–1.95], *P* = 0.001; additive model: adjusted OR = 1.50, 95% CI [1.27–1.78], *P* < 0.001) and IFIH1 rs10930046-C allele (additive model: adjusted OR = 1.26, 95% CI [1.07–1.49], *P* = 0.005) were associated with chronic hepatitis C (CHC). And the risk of CHC increased in people carrying more unfavorable genotypes (rs2074158-AG/GG or rs10930046-CC), with the chronic rates for genotypes number from zero to two in 60.69%, 57.33%, and 85.93%, respectively (adjusted OR = 3.64, 95% CI [2.18–6.08]; *P* < 0.001). Genetic polymorphism of IFIH1 and DHX58 may be related to CHC in the Chinese Han population. Furthermore, the risk of CHC increases as the number of unfavorable genotypes carried by the HCV-infected person increases. IFIH1 rs10930046, DHX58 rs2074158, age, ALT, and AST levels were all independent predictors of CHC.

## Introduction

As estimated by the World Health Organization, 71 million people are under chronic HCV infection worldwide (Stasi, Silvestri & Voller, 2020). More than half of individuals living with HCV reside in Asia and the Pacific area (29.6 million), with China having the largest HCV epidemic (9.8 million) (Hill, Nath & Simmons, 2017). There are two manifestations of HCV infection: acute infection (the virus clears within six months) and chronic infection (the immune system is unable to clear the virus) (Chigbu et al., 2019). Approximately 30% of patients could achieve spontaneous clearance during the acute infection period (Chigbu et al., 2019), whereas the remaining 70% would develop a chronic infection. HCV genotype 1b is more common in hepatitis C patients, and this study population was no exception. After recurrent liver damage, chronic HCV infection frequently results in poor life outcomes such as liver failure, cirrhosis, and hepatocellular carcinoma (HCC) (Chigbu et al., 2019). HCV infection is an urgent problem to be solved in reducing deaths from liver-related diseases, which account for approximately 700,000 deaths annually (Rowe, 2017; Stasi, Silvestri & Voller, 2020). However, more resources and innovation are needed to achieve the 2030 elimination target (Thomas, 2019; Thomas, 2020).

HCV is a member of the Flaviviridae family, which is a positive-sense single-stranded RNA virus with a highly structured genome that is 9.6 kb in length (Adams, Pirakitikulr & Pyle, 2017). During the replication process, viral RNA polymerase generates a negative RNA that encodes a single polyprotein that is the target of the host’s innate immune system (Adams, Pirakitikulr & Pyle, 2017). The recognition of viral infection and induction of the innate antiviral immune response is activated by the recognition of pathogen-associated molecular patterns present in the viral genome through binding to pattern recognition receptors (PRRs). Following that, a series of signal cascades are activated, resulting in the production and secretion of type I interferons for viral defense and immune regulation (Li et al., 2014; Ireton, Wilkins & Gale, 2017). Retinoic acid-inducible gene-I-like receptor (RLRs) is the main PRRs for RNA viruses (International Committee on Taxonomy of Viruses, 2000; Adams, Pirakitikulr & Pyle, 2017; Chigbu et al., 2019). RLRs contain retinoic acid-inducible gene I (RIG-I), melanoma differentiation antigen 5 (MDA5), and laboratory of genetics and physiology 2 (LGP2), belonging to the RNA helicase superfamily (Loo & Gale, 2011), which are encoded by *DHX58*, *IFIH1*, and *DHX58* respectively, and expressed in most cell types in the human body and play an important role in the immune response to RNA virus infection (Ireton, Wilkins & Gale, 2017). Hence, the ability to eliminate HCV may be closely related to RLRS.

Host factors like gender and genetic variants, as well as viral factors such as HCV RNA levels, have been related to clearance and chronicity. Studies have associated higher chronicity rates with allele polymorphisms of Human leukocyte antigen class II and Interleukin 28 (Rauch et al., 2010; Lingala & Ghany, 2015). This further suggests that the host immune system may play a key role in viral clearance. However, there is limited knowledge about these variants according to current research (Vergara et al., 2019). Therefore, it is necessary to study the relationship between RLRs family gene polymorphisms and HCV chronic infection.

## Materials & Methods

### Study participants

This study included three types of high-risk groups of HCV infection, including a person who uses drugs from the compulsory rehabilitation center of Nanjing Public Security Bureau from May 2006 to December 2006 (*N* = 311), hemodialysis patients from nine hospital dialysis centers in Jiangsu Province from October 2008 to December 2009 (*N* = 184), former paid-blood donors from nine natural administrative villages in a certain area of Zhenjiang from April 2011 to April 2015 (*N* = 1095). All of the research objects had HCV antibody positive for more than 6 months and voluntarily signed an informed consent form. The inclusion criteria of the research subjects are as follows: (1) The patient was diagnosed with HCV by an experienced doctor based on clinical symptoms and strict compliance with the international standard biochemical examination indicators; (2) patients with complete baseline information (age, gender, and biochemical markers) and HCV antibody and viral load information. The exclusion criteria of the research subjects are as follows: (1) Han population patients under the age of 18; (2) patients with interferon treatment history; (3) patients co-infected with HBV and HIV; (4) patients who suffer from autoimmune disease or malignant tumor; (5) patients with other types of liver disease (including liver-related genetic diseases such as hepatolenticular degeneration which is a single-gene hereditary liver disease). All Patients were divided into categories based on their HCV antibodies and viral load. HCV antibody-positive and HCV RNA-negative spontaneous HCV clearance were identified. Persistent HCV infection was defined as anti-HCV positive and HCV-RNA positive. The study was conducted following the Declaration of Helsinki, and the protocol was approved by the Ethics Committee of Nanjing Medical University (2017445).

### Data and blood sample collection

The patient’s demographic and clinical baseline data are collected through the electronic medical record system. Professional medical personnel who had undergone standardized training through formal procedures collected 5 ml of EDTA anticoagulant blood from each participant. Blood samples were centrifuged and separated into red blood cells, serum, and white blood cell parts within 24 h and stored at −20 °C. The serum samples were used to detect HCV antibodies and biochemical indicators of liver function (alanine aminotransferase and aspartate aminotransferase).

### SNP selection and genotyping

First, the genotype information of the target genes of the Chinese Han population was downloaded from the 1,000 Genome Project website (http://www.1000genomes.org/) Single-nucleotide polymorphisms (SNPs) were selected with the help of Haploview 4.2 software. r2 (linkage disequilibrium coefficient) higher than 0.8 (Kim & Kirkpatrick, 2009), and MAF ≥0.1 is defined as tag SNP. Secondly, the potential functions of tag SNPs were explored based on bioinformatics databases (NCBI, GTEx Portal, HaploReg v4.1 and SNP Function Prediction, *etc*.). Combining GTEx Portal to analyze quantitative traits of different genotypes of SNPs with the SNP Function Prediction database to see if SNPs have miRNA binding functions, for example, allows us to further investigate potential functions. Thirdly, the search results were explored for potential SNPs in the existing research literature. Previous studies have shown that mutations at *IFIH1* rs10930046 and *DHX58* rs2074158 rs2074160 were associated with infectious and autoimmune diseases (Zhang et al., 2018; Yao et al., 2021), both of which were closely related to the body’s immune function. Therefore, we select these three interesting SNPs for this study for further analysis.

To extract genomic DNA from white blood cells, the phenol-chloroform extraction method was used, and the NanoDrop2000 was utilized to detect DNA purity and concentration. Samples with a concentration of TaqMan allelic discrimination assay on the ABI 7900HT sequence detection system were used to analyze the gene polymorphism. The information on primers and probes is shown in Table S1. The operator was uninformed of the participants’ clinical data. Each 384-well format is programmed with two blank controls. The polymerase chain reaction (PCR) program was set at 50 °C for 2 min, 95 °C for 10 min, 95 °C for 45 cycles for 15 s, and 60 °C for 1 min. 10% of the samples were randomly selected to repeat experiments, and 100% consistency was achieved. The success rates of SNPs genotyping were above 90%.

### Statistical analysis

Study participants’ characteristics were described as the mean ± standard deviation or as counts and proportions. Differences in selected variables were compared between HCV spontaneous clearance group and the persistent infection group using Student’s *t*-test or Chi-square test. The association between candidate SNP genotypes and CHC risk was investigated using multivariate logistic regression analysis, and the results were given as odds ratios and 95% confidence intervals. In this study, the dominant (heterozygote + mutational homozygote *versus* wild homozygote), recessive (heterozygote *versus* mutational homozygote + wild homozygote), and additive (wild homozygote *versus* heterozygote *versus* mutational homozygote) genetic models were utilized to explore the exact patterns in which a genetic variation works. The trend analysis was assessed with the Cochran–Armitage trend test. HaploView was used to calculate LD parameters (*i.e.,* r2 and D’), and PHASE software (version 2.1; UW TechTransfer Digital Ventures, University of Washington, Seattle, WA, USA) was used to reconstruct the haplotype block and estimate the frequencies. Then, stratified analysis was performed to explore the underlying factors which may influence the outcome. Cochran’s Q test was used to estimate the heterogeneity between the subgroups. All statistical analyses were performed by Stata (version 12.0, STATA Corp, College Station, TX, USA). All tests were two-sided, *P* values < 0.05 were considered statistically significant, and multiple comparisons were corrected by FDR.

## Results

### Demographic and clinical characteristics of participants

A total of 1,590 patients were enrolled, including 590 spontaneous clearance patients (215 males, 375 females) and 1,000 persistent infection cases (337 males, 663 females). The mean age of the persistent infection group (54.03 ± 11.58) was higher than the spontaneous clearance group (49.66 ± 13.51) (*P* < 0.001). In addition, the serum levels of alanine aminotransferase (ALT) and aspartate transaminase (AST) were higher in the persistent infection group (*P* < 0.001). The basic characteristics of the two groups were presented in Table 1.

**Table 1:** Baseline characteristics of HCV spontaneous clearance and persistent infection populations.

**Variables**	**Spontaneous HCV clearance (%)** ***N* = 590**	**Persistent HCV** **infection (%)** ***N* = 1000**	**P-value**
Age (years)	49.66 ± 13.51	54.03 ± 11.58	**<0.001**
≤50	162 (27.46)	136 (13.60)	
>50	428 (72.54)	864 (86.40)	
Gender			0.267
Male	215 (36.44)	337 (33.70)	
Female	375 (63.56)	663 (66.30)	
ALT (U/L)	22 (14,37)	27 (16,50)	**<0.001**
<40	460 (77.97)	605 (60.50)	
≥40	130 (22.03)	395 (39.50)	
AST (U/L)	26 (19,35)	32 (23,49)	**<0.001**
<40	466 (78.98)	566 (56.60)	
≥40	124 (21.02)	434 (43.40)	**<0.001**

**Notes.**

Abbreviations ALT alanine transaminase AST aspartate aminotransferase

Bold font indicates that the data is statistically significant.

### Association of candidate genes polymorphisms with HCV chronicity

Table 2 shows the genotype distributions of the three potential SNPs (*IFIH1* rs10930046, *DHX58* rs2074158, rs2074160) and their relationships with HCV chronicity. After adjusting for age, gender, serum ALT, and AST levels, multivariate logistic regression showed that *DHX58* rs2074158 (Dominant model: adjusted OR = 1.53, 95% CI [1.20–1.95], *P* = 0.001; Additive model: adjusted OR = 1.50, 95% CI [1.27–1.78], *P* < 0.001) and *IFIH1* rs10930046 (Additive model: adjusted OR =1.26, 95% CI [1.07–1.49], *P* = 0.005) were significantly associated with CHC risk. A significant correlation was found between candidate SNPs and CHC risk after FDR correction (*P* = 0.003 and 0.008, respectively) (Table S2). Subsequently, the combined impact of rs2074158 and rs10930046 was evaluated depending on the number of unfavorable genotypes (Table 3). The results demonstrated that the risk of CHC increased with the number of unfavorable rs2074158-AG/GG and rs10930046-CC genotypes increased (P_trend_<0.001). Compared with patients not carrying risk genotypes, CHC patients with two risk alleles have a 3-fold higher risk of chronic disease than those without risk genotypes (adjusted OR = 3.64, 95% CI [2.18–6.08], *P* < 0.001).

**Table 2:** Association of selected SNPs with chronic hepatitis C.

**SNPs** **(genotype)**	**Spontaneous HCV clearance** ***N* = 590**	**Persistent** **HCV infection** ***N* = 1000**	***OR*** **(95% CI)**	***P-value***
rs2074158				
AA	429 (75.53)	650 (67.22)	1.00	–
AG	104 (18.31)	153 (15.82)	1.03 (0.77–1.37)	0.865
GG	35 (6.16)	164 (16.96)	2.99 (2.01–4.44)	**<0.001**
Dominant model				
AA	429 (75.53)	650 (67.22)	1.00	–
AG/GG	139 (24.47)	317 (32.78)	1.53 (1.20–1.95)	**0.001**
Recessive model				
AA/AG	533 (93.84)	803 (83.04)	1.00	–
GG	35 (6.16)	164 (16.96)	2.97 (2.03–4.46)	**<0.001**
Additive model			1.50 (1.27–1.78)	**<0.001**
rs10930046				
TT	385 (67.54)	594 (63.26)	1.00	–
TC	150 (26.32)	211 (22.47)	0.87 (0.67–1.12)	0.285
CC	35 (6.14)	134 (14.27)	2.32 (1.54–3.48)	**<0.001**
Dominant model				
TT	385 (67.54)	594 (63.26)	1.00	–
TC/CC	185 (32.46)	345 (36.74)	1.14 (0.91–1.44)	0.259
Recessive model				
TT/TC	535 (93.86)	805 (85.73)	1.00	–
CC	35 (6.14)	134 (14.27)	2.41 (1.63–3.65)	**<0.001**
Additive model			1.26 (1.07–1.49)	**0.005**
rs2074160				
GG	446 (79.93)	762 (80.55)	1.00	–
GA	98 (17.56)	152 (16.07)	0.96 (0.72–1.29)	0.793
AA	14 (2.51)	32 (3.38)	1.70 (0.87–3.34)	0.121
Dominant model				
GG	446 (79.93)	762 (80.55)	1.00	–
GA/AA	112 (20.07)	184 (19.45)	1.05 (0.80–1.37)	0.748
Recessive model				
GG/GA	544 (97.49)	914 (96.62)	1.00	–
AA	14 (2.51)	32 (3.38)	1.72 (0.89–3.45)	0.115
Additive model			1.10 (0.88–1.38)	0.410

**Notes.**

Abbreviations HCV hepatitis C virus OR odds ratio CI confidence interval

The *P* value of persistent HCV infection versus spontaneous HCV clearance was calculated based on the logistic regression model, adjusted by age, gender, ALT and AST.

Bold font indicates that the data is statistically significant.

**Table 3:** Association of the number of dangerous genotypes with chronic hepatitis C.

**Risk alleles**	**Spontaneous HCV clearance** ***N* = 590**	**Persistent** **HCV infection** ***N* = 1000**	**OR, 95% CI**	**P-value**	
0	401 (72.78)	619 (67.95)	1.00	–	
1	131 (23.77)	176 (19.32)	0.94 (0.72–1.23)	0.643	
2	19(3.45)	116 (12.73)	3.64 (2.18–6.08)	**<0.001**	
Trend				**P** ^**a**^ **=2.929 × 10-5**	
0	401 (72.78)	619 (67.95)	1.00	–	
1–2	150 (27.22)	292 (32.05)	1.30 (1.02–1.66)	**<0.001**	

**Notes.**

Abbreviations HCV hepatitis C virus OR odds ratio CI confidence interval

The risk alleles is the number of unfavorable genotypes (rs2074158-AG/GG, rs10930046-CC).

The *P* value of persistent HCV infection versus spontaneous HCV clearance was calculated based on the logistic regression model, adjusted by age, gender, ALT and AST.

P^a^-value was from Cochran–Armitage trend test.

Bold font indicates that the data is statistically significant.

Two-locus haplotypes consisting of rs2074158 and rs10930046 variant alleles were reconstructed using HaploView software and PHASE software (Table 4). Compared with participants with the most frequent AT haplotype, those with the AC haplotype showed significantly higher persistent infection risk (OR = 1.295, 95% CI [1.070–1.566], *P* = 0.008), while GT and GC haplotypes showed no significant associations (*P* > 0.05).

**Table 4:** Haplotype analysis of rs2074158 and rs10930046 in the study population.

**Haplotype**	**Spontaneous HCV clearance**	**Persistent HCV infection**	**OR (95% CI)**	***P***
**(rs2074158–rs10930046)**				
AT	841	1322	1	1
AC	213	462	1.295 (1.070–1.566)	0.008
GT	119	199	1.140 (0.888–1.464)	0.304
GC	7	17	1.664 (0.662–4.186)	0.279

### Stratified analysis

According to the combined variant genotypes of two SNPs (rs2074158 and rs10930046), a stratified analysis was performed to assess whether genetic associations are consistent across subgroups and explore how potential confounders may disorder the observed genetic (Table 5). The combined effects of these independent SNPs and CHC were more significant among female patients (adjusted OR = 1.49, 95% CI [1.10–2.03], *P* = 0.011) and patients whose serum AST level was less than 40 U/L (adjusted OR = 0.73, 95% CI [0.55–0.97] *P* = 0.032) and more than 40U/L (adjusted OR = 1.68, 95% CI [1.10–2.57], *P* = 0.016). AST subgroup was significant heterogeneity with CHC risk, but the remaining subgroups were not. Therefore, the interaction between AST levels and the combination of these two SNPs was investigated, and the findings revealed that the interaction was not statistically significant (Table S3).

**Table 5:** Stratified analysis on combined variant genotypes and chronic hepatitis C.

**Subgroups**	**Spontaneous HCV clearance**		**Persistent HCV infection**		***OR*** **(95% CI)**	***P*** ^a^	***P*** ^b^
	**CHC risk** ^c^ **,0**	**CHC risk** ^c^ **,1-2**	**CHC risk** ^c^ **,0**		**CHC risk** ^c^ **,1-2**			
Age (years)							0.828
<50	109 (71.24)	44 (28.76)	86 (68.25)		40 (31.75)	1.230 (0.729–2.076)	0.437	
≥50	292 (73.97)	106 (26.63)	533 (67.90)		252 (32.10)	1.315 (0.998–1.739)	0.052	
Gender							0.138
Male	140 (69.65)	61 (30.35)	213 (70.76)		88 (29.24)	1.013 (0.674–1.525)	0.949	
Female	261 (74.57)	89 (25.43)	406 (66.56)		204 (33.44)	1.492 (1.096–2.031)	0.011	
ALT (U/L)							0.446
<40	304 (70.86)	125 (29.14)	356 (65.32)		189 (34.68)	0.881 (0.667–1.161)	0.371	
≥40	97 (79.51)	25 (20.49)	263 (71.86)		103 (28.14)	1.099 (0.705–1.712)	0.677	
AST (U/L)							0.015
<40	309 (71.20)	125 (28.80)	355 (69.47)		156 (30.53)	0.734 (0.553–0.974)	0.032	
≥40	92 (78.63)	25 (21.37)	264 (66.00)		136 (34.00)	1.683 (1.100–2.573)	0.016	

**Notes.**

This table illustrates the effects of adverse alleles on HCV chronicity between groups of confounding factors.

Abbreviations ALT alanine transaminase AST aspartate aminotransferase HCV hepatitis C virus OR odds ratio CI confidence interval

a The *P*-value of persistent HCV infection versus spontaneous HCV clearance was calculated by the logistic regression model, adjusted by sex, age, ALT, and AST.

b The *P*-value was the result of the heterogeneity test.

c CHC risk: The number of unfavorable genotypes (0 vs 1–2).

### Bioinformatics analysis of *IFIH1*-*DHX58* SNPs

**Figure 1:**
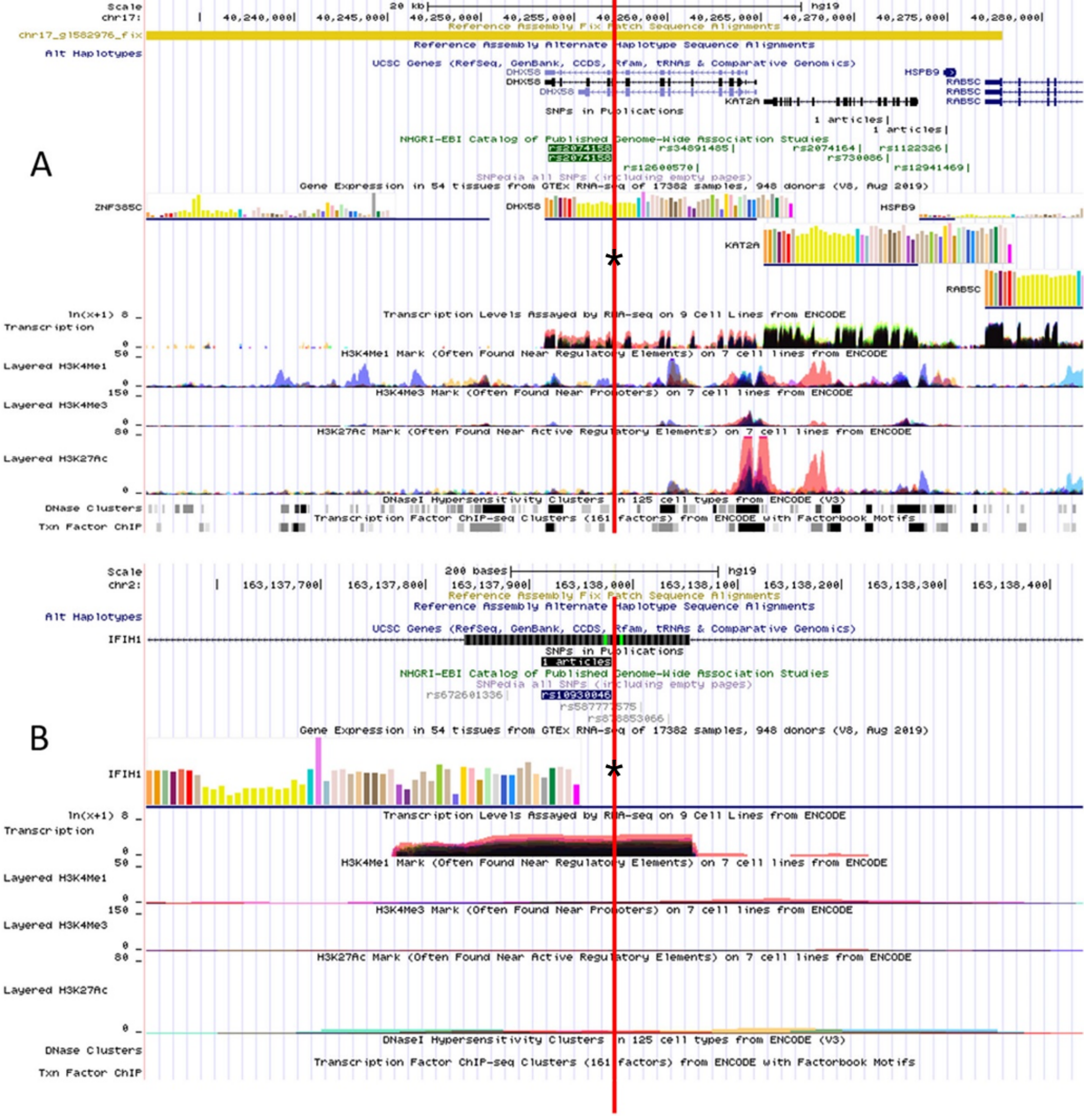
Functional annotation of (A) DHX58 rs2074158 and (B) IFIH1 rs10930046 based on ENCODE data in UCSC functional website. Note: the red line with an asterisk (*) in the middle of the picture marks the location of (A) DHX58 rs2074158 and (B) IFIH1 rs10930046.

**Figure 2:**
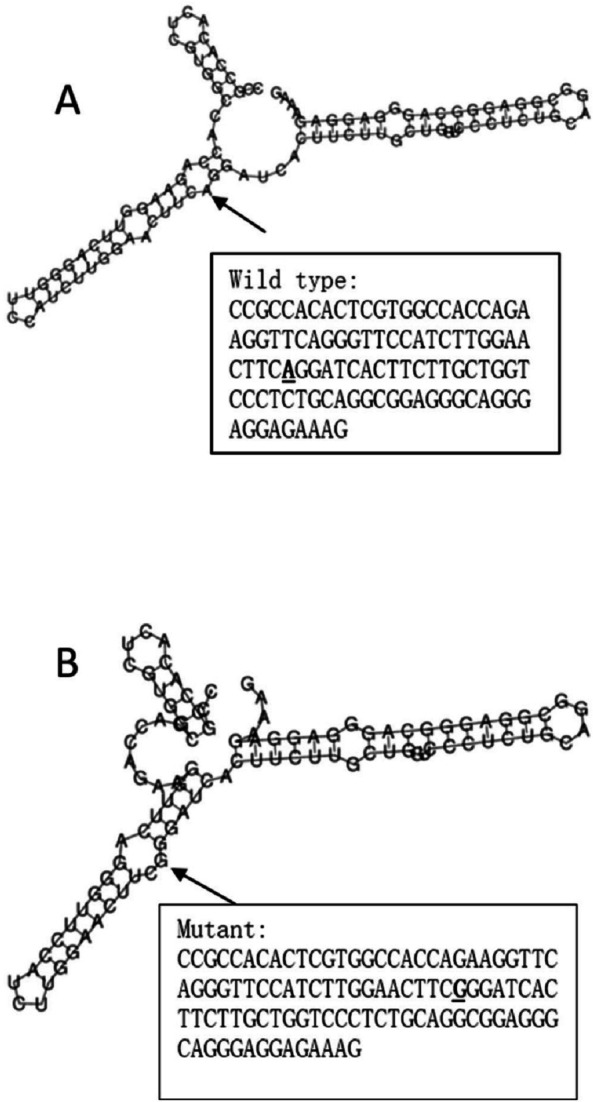
The influence of rs2074158 on mRNA centroid secondary structures of DHX58. Changes in the local structure were illustrated by the RNAfold Web Server. The arrow indicates the position of the mutation (50 bases upstream and 50 bases downstream from the mutation). The minimum free energy of the mRNA centroid secondary structure (a structure with minimal base pair distance) for wild type and mutant rs10930046 were estimated to be −42.60 kcal/mol (A) and −43.20 kcal/mol (B), respectively. The wild-type and mutant-type sequences are listed on the right. The bold and underlined font indicates the nucleotide difference between the wild and mutant allele.

**Figure 3:**
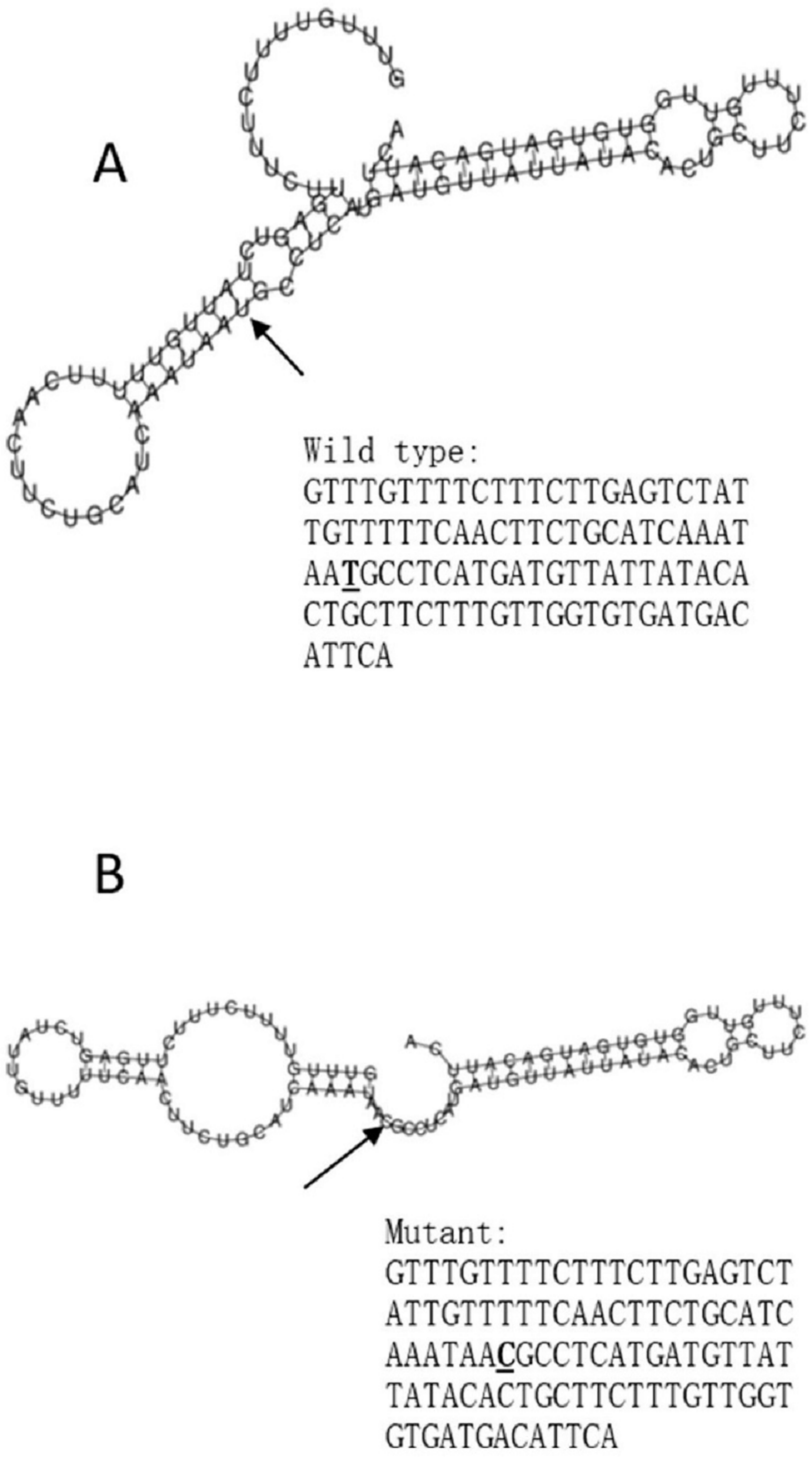
The influence of rs10930046 on mRNA centroid secondary structures of IFIH1. Changes in the local structure were illustrated by the RNAfold web server. The arrow indicates the position of the mutation (50 bases upstream and 50 bases downstream from the mutation). The minimum free energy of the mRNA centroid secondary structure (a structure with minimal base pair distance) for wild type and mutant rs10930046 were estimated to be −18.0 kcal/mol (A) and −16.50 kcal/mol (B), respectively. The wild-type and mutant-type sequences are listed on the right. The bold and underlined font indicates the nucleotide difference between the wild and mutant allele.

The RegulomeDB rank for rs2074158 and rs10930046 were 1f and 7, respectively. It indicates that rs2074158 may be related to the potential function, such as expression Quantitative Trait Loci, TF binding, or DNase peak. The results of the UCSC database suggest that the positive SNP exerts its effect on HCV infection by affecting the transcription function (Figs. 1A, 1B). To further study the impact of mutations on transcription changes. The RNA fold web server was also used to further predict the secondary structure of mRNA and obtain its minimum free energy. The changes in mRNA structure caused by the rs2074158 polymorphism are depicted in Fig. 2. The MFE of the centroid mRNA secondary structure of the mutant G allele is −43.2 kcal/mol, which is higher than that of the wild-type A allele. The changes in mRNA structure due to the rs10930046 polymorphism are shown in Fig. 3. The MFE of the centroid mRNA secondary structure of the mutant C allele is −16.5 kcal/mol, which is lower than that of the wild-type T allele.

## Discussion

HCV is a major global threat. Until 2040, deaths from chronic hepatitis are projected to exceed the combined mortality associated with HIV infection, tuberculosis, and malaria (Foreman et al., 2018). Therefore, it is necessary to study the mechanism of HCV chronic infection.

RIG-I-like receptors play a very important role in the establishment of innate immunity against viruses. Our research showed that the rs2074158 polymorphism of the *DHX58* gene and the rs10930046 polymorphism of the *IFIH1* gene are risk factors for chronic HCV infection. A GWAS analysis also revealed that the rs76398191 polymorphism of the ARL5B gene is related to the chronicity of HCV infection (Vergara et al., 2020). Variants in MHC, IFNL4–IFNL3, and GPR158 increase the odds of HCV clearance in patients of European and African ancestry (Vergara et al., 2019). The polymorphism of rs3747517 in the *IFIH1* gene is also related to the chronicity of HCV (Hoffmann et al., 2015). These suggest that genetic variants correlated with interferon may influence the chronicity of HCV possibly through regulation of gene expression. The outcome of HCV infection (*i.e.,* the persistence of the virus) and the presentation and extent of liver disease are the results of a complex interplay between the virus and the host immune response (Gremion & Cerny, 2005). Several studies have shown that the HLA gene, TLR gene, and NK gene variants are related to HCV chronic disease (Wang et al., 2011; Spengler et al., 2013).

Regarding the connection between both the *IFIH1* rs10930046-C mutation and chronic HCV infection, MDA5 is a dsRNA helicase that is encoded by the *IFIH1* gene. MDA5 binds to the viral RNA in the cytoplasm to activate the downstream immune cascade and release cytokines to exert the host’s antiviral effect. Even in cancer cells, MDA5 can interact with cellular RNA to induce an immune response (Dias Junior, Sampaio & Rehwinkel, 2019). MDA5 is the main PRR that recognizes HCV. Cellular experiments showed that the activation of interferon caused by HCV infection mainly depends on the induction of MDA5 (Cao et al., 2015). Several studies have shown that genetic polymorphisms of *IFIH1* are associated with autoimmune-related diseases (Smyth et al., 2006; Looney et al., 2015) and AGS (a rare progressive encephalopathy) (Oda et al., 2014). Some studies have found that *IFIH1* rs10930046 is related to the occurrence of systemic lupus erythematosus and diabetes (Rodríguez et al., 2015; Silva et al., 2016), which can infer that *IFIH1* rs10930046 can affect the immune function disorder of the body, and then lead to the occurrence of the disease. From this point of view, chronic HCV infection may be a manifestation of immune disorders. Combined with the bioinformatics analysis of UCSC and RNA fold Web server, the rs10930046 mutation may affect the transcription function of *IFIH1* and then change the secondary structure of mRNA and affect MDA5 to exert its host immune and antiviral response function.

However, the effective antiviral response mediated by MDA5 mainly depends on LGP2. LGP2 is an ATP-dependent RNA helicase. It was found that the *DHX58* rs2074158-G mutation is related to chronic HCV infection. LGP2 can have antiviral effects in several ways, including upregulating apoptosis regulatory genes to increase cell apoptosis during viral infection (Takahashi et al., 2020). Different RLR receptors have different recognition capabilities and signal characteristics for RNA (Yoneyama et al., 2005). The common viral antagonism supports a positive role for LGP2 and a connection with MDA5 (Rodriguez, Bruns & Horvath, 2014). Studies have shown that LGP2 acts as a negative feedback regulator of antiviral signals, which may be due to LGP2 having a stronger affinity for viral RNA than MDA5 (Rothenfusser et al., 2005). In addition, since LGP2 lacks a signal transduction domain, it can play a role in viral infection by cooperating with MDA5, and it can act as a concentration-dependent switch between MDA5-specific enhancement and interference. LGP2 can regulate the activation ability of MDA5 through concentration changes, which in turn affects the occurrence of downstream cascades (Rodriguez, Bruns & Horvath, 2014). Studies have shown that an SNP mutation in the *DHX58* gene disrupts the LGP2-PACT interaction, leading to the loss of LGP2-mediated MDA5 signaling regulation (Sanchez David et al., 2019). Based on bioinformatics, we found that rs2074158-G is a missense mutation with a RegulomeDB rank of 1f, which is predicted to affect TF binding and DNase peak. Based on UCSC and ENCODE database analysis, rs2074158 is located near the active promoter element (H3K4Me1) in 7 cell lines. Possibly pathogenic SNPs are enriched in enhancer and regulatory element areas, particularly in the H3K4Me1 region, indicating that these alterations may impact disease progression through the regulation of gene transcription and expression. In conclusion, SNP rs2074158 may affect the function of the *DHX58* gene through several possible mechanisms, including regulation of gene expression, thereby affecting the host immune function by interfering with the effective antiviral response of MDA5, and ultimately affecting the outcome of HCV infection.

At the same time, the chronicity of HCV is associated with the increase in the number of unfavorable alleles of *DHX58* rs2074158 and *IFIH1* rs10930046 (OR = 1.30). This further indicates that the chronicity of HCV is contributed by the weakened antiviral response of LGP2 and MDA5. This combined effect was statistically significant between gender (female) and different subgroups of AST. The test of heterogeneity between genders was not statistically significant. There is statistical significance between different AST levels, and the effect is opposite between different AST subgroups. In people with AST<40 U/L, carrying unfavorable alleles is a protective factor for HCV chronicity (OR = 0.73), while it is a risk factor in people with AST ≥40 U/L (OR = 1.68). It suggested that the synergistic effects of LGP2 and MDA5 may be inconsistent in individuals with different levels of AST, resulting in an antiviral response that is the opposite. The activity of AST is known to be involved in the biosynthesis of mitochondrial amino acids (Kirsch et al., 1984; Chang, Liu & Lee, 2019). Animal experiments show that the liver injury group showed higher mitochondrial oxygen consumption, higher enzyme activity, and higher ATP levels. AST also effectively inhibits the inflammatory response in the rat brain and down-regulates the expression of P2X7R (ATP-gated purinergic receptor). All these suggest that AST dysfunction and energy metabolism may have a synergistic interaction (Guazzelli et al., 2019; Er et al., 2020; Wang et al., 2020) which in turn affects the ATP-dependent LGP2 and MDA5 to play a synergistic antiviral effect, but the specific mechanism is not clear and further research is needed.

This study also has some limitations. Firstly, this study was analyzed in a specific population, so the research results may not apply to other populations. Secondly, due to the limitation of the number of patients, manpower, and material resources, the selection of SNPs is restricted and we lacked detailed data on viral load and HCV genotypes which is to be aware of in the next study. Finally, there is no method for validating animal experiments in this paper. In the future, we plan to perform animal and cell research to confirm our results.

## Conclusions

In conclusion, genetic polymorphism of *IFIH1* and *DHX58* may be related to CHC in the Chinese Han population. Furthermore, the risk of CHC increases as the number of unfavorable genotypes carried by the HCV-infected person increases. *IFIH1* rs10930046, *DHX58* rs2074158, age, ALT, and AST levels were all independent predictors of CHC.

##  Supplemental Information

10.7717/peerj.14740/supp-1 Supplemental Information 1 Raw data

10.7717/peerj.14740/supp-2 Supplemental Information 2 Codebook for raw data

10.7717/peerj.14740/supp-3 Supplemental Information 3 Primers sequences of selected SNPs in IFIH1 and DHX58 gene MAF and *P*_*H*_*WE* were calculating from 554 East Asian population in 1000 Genomic Project using *PLINK* (v.1.90).

10.7717/peerj.14740/supp-4 Supplemental Information 4 Associations of IFIH1-DHX58 SNPs with HCV infection outcomes in dominant and additive models of multivariable analyses *P*_*F*_*DR* is expressed as the *P* value after multiple corrections by the FDR method

10.7717/peerj.14740/supp-5 Supplemental Information 5 Interaction of rs2074158& rs10930046 with serum AST level

10.7717/peerj.14740/supp-6 Supplemental Information 6 Allele frequencies of studied SNPs in different populations from the NCBI dbSNP database

## Additional Information and Declarations

### Data Availability

The following information was supplied regarding data availability: Raw data is available in the Supplemental Files.
