# Supplementary material for: Genetic variants of IFIH1 and DHX58 affect the chronicity of hepatitis C in the Chinese Han population
Source: PeerJ. 2023 Jan 30;11:e14740. doi: 10.7717/peerj.14740 (PMC9893905; doi:10.7717/peerj.14740)
Supplement: Supplemental Information 3 — MAF and PHWE were calculating from 554 East Asian population in 1000 Genomic Project using PLINK (v.1.90). [file peerj-11-14740-s003.docx]

**Table S1. Primers sequences of selected SNPs in IFIH1 and DHX58 gene**

| **SNPs** |  | **Primers sequences（5’-3’）** | **MAF** | ***P_HWE_*** |
| --- | --- | --- | --- | --- |
| rs10930046 | Primers | F: GCTGTTAGTCCCAGTATCTGAGGGAA | 0.125 | 0.841 |
| (T>C) |  | R: ATCACACCAACAAAGAAGCAGTGT |  |  |
|  | Probes | FAM- CATCAAATAATGCCTCATG-MGB  HEX- CATCAAATAACGCCTCAT-MGB |  |  |
| rs2074158 | Primers | F: CCTCATGTACCAGCCTCTTTCTC | 0.188 | 1.000 |
| (A>G) |  | R: CCAGAAGGTTCAGGGTTCCA |  |  |
|  | Probes | FAM- CAAGAAGTGATCCGGAA-MGB |  |  |
|  |  | HEX- TGATCCAGAAGTTCCA-MGB |  |  |
| rs2074160 | Primers | F: CTCTTCCAGGTACAAATGATCTTAGTTC | 0.167 | 0.336 |
| (G>A) |  | R: CGCCCGCTTGGTCAAG |  |  |
|  | Probes | FAM- CCCCAGATCCGGG-MGB |  |  |
|  |  | HEX-ACCCCAGATCCAGG-MGB |  |  |
| MAF and *P_HWE_* were calculating from 554 East Asian population in 1000 Genomic Project using *PLINK* (v.1.90) | | | | |
