# Supplementary material for: Genetic variants of IFIH1 and DHX58 affect the chronicity of hepatitis C in the Chinese Han population
Source: PeerJ. 2023 Jan 30;11:e14740. doi: 10.7717/peerj.14740 (PMC9893905; doi:10.7717/peerj.14740)
Supplement: Supplemental Information 4 — PFDR is expressed as the P value after multiple corrections by the FDR method [file peerj-11-14740-s004.docx]

**Table S2. Associations of** **IFIH1-DHX58 SNPs with HCV infection outcomes in dominant and additive models of multivariable analyses**

| **SNPs** | **Dominant model** | | **Dominant model** | | **Additive model‡** | |
| --- | --- | --- | --- | --- | --- | --- |
|  | ***P*** | ***P_FDR_*** | ***P*** | ***P_FDR_*** | ***P*** | ***P_FDR_*** |
| Rs10930046 (T>C) | 0.259 | 0.389 | <0.001 | <0.001 | 0.005 | 0.008 |
| Rs2074158 (A>G) | 0.001 | 0.003 | <0.001 | <0.001 | <0.001 | <0.001 |
| Rs2074160 (G>A) | 0.748 | 0.748 | 0.115 | 0.115 | 0.410 | 0.410 |

*P_FDR_* is expressed as the P value after multiple corrections by the FDR method
