# Supplementary material for: Genetic variants of IFIH1 and DHX58 affect the chronicity of hepatitis C in the Chinese Han population
Source: PeerJ. 2023 Jan 30;11:e14740. doi: 10.7717/peerj.14740 (PMC9893905; doi:10.7717/peerj.14740)
Supplement: Supplemental Information 5 [file peerj-11-14740-s005.docx]

**Table S3. Interaction of rs2074158& rs10930046 with serum AST level**

| **CHC risk AST** | | **Spontaneous HCV clearance** | **Persistent HCV infection** | **OR** | **P** | **P(interaction)** |
| --- | --- | --- | --- | --- | --- | --- |
| 1-2 | 1 | 136(46.58) | 25(16.67) | 3.51(2.14-5.75) | 0.000 | 0.0612 |
| 1-2 | 0 | 156(53.42) | 125(83.33) | 1.13(0.85-1.50) | 0.411 |  |
| 0 | 1 | 264(42.65) | 92(22.94) | 1.81(1.25-2.62) | 0.002 |  |
| 0 | 0 | 355(57.35) | 309(77.06) | 1 | - |  |
