# Supplementary material for: Genetic variants of IFIH1 and DHX58 affect the chronicity of hepatitis C in the Chinese Han population
Source: PeerJ. 2023 Jan 30;11:e14740. doi: 10.7717/peerj.14740 (PMC9893905; doi:10.7717/peerj.14740)
Supplement: Supplemental Information 6 [file peerj-11-14740-s006.docx]

**Table S4. Allele frequencies of studied SNPs in different populations from the NCBI dbSNP database**

| **Population** | **IFIH1-rs10930046** | | | **DHX58-rs2074158** | | | **DHX58-rs2074160** | | |
| --- | --- | --- | --- | --- | --- | --- | --- | --- | --- |
|  | **Sample**  **Size** | **Ref**  **Allele (T)** | **Alt**  **Allele (C)** | **Sample**  **Size** | **Ref**  **Allele (T)** | **Alt**  **Allele (C)** | **Sample**  **Size** | **Ref**  **Allele (C)** | **Alt**  **Allele (T)** |
| European | 246710 | 0.989 | 0.011 | 227242 | 0.832 | 0.168 | 26570 | 0.984 | 0.016 |
| African | 9388 | 0.624 | 0.378 | 9576 | 0.304 | 0.697 | 3314 | 0.788 | 0.212 |
| African American | 9086 | 0.628 | 0.372 | 9282 | 0.309 | 0.691 | 3204 | 0.790 | 0.210 |
| Asian | 6664 | 0.857 | 0.144 | 3612 | 0.864 | 0.137 | 168 | 0.810 | 0.190 |
| East Asian | 4786 | 0.865 | 0.135 | 2312 | 0.871 | 0.129 | 112 | 0.857 | 0.143 |
| South Asian | 358 | 0.983 | 0.017 | 5018 | 0.790 | 0.210 | 98 | 0.930 | 0.070 |
| Other | 20658 | 0.951 | 0.049 | 18474 | 0.809 | 0.191 | 4344 | 0.967 | 0.033 |
| Total | 290036 | 0.966 | 0.034 | 272546 | 0.812 | 0.188 | 35250 | 0.962 | 0.038 |
